# Two coastal Pacific evergreens, *Arbutus menziesii*, Pursh. and *Quercus agrifolia*, Née show little water stress during California's exceptional drought

**DOI:** 10.1371/journal.pone.0230868

**Published:** 2020-04-02

**Authors:** Alexander I. Chacon, Alexander Baer, James K. Wheeler, Jarmila Pittermann

**Affiliations:** Department of Ecology and Evolutionary Biology, University of California, Santa Cruz, California, United States of America; University of British Columbia, CANADA

## Abstract

California's coastal climate is characterized by rainy winters followed by a dry summer season that is supplemented by frequent fog. While rising temperatures and drought caused massive tree mortality in central California during the 2011–2015 extreme drought, dying trees were less common in the central coast region. We hypothesized that cooler, maritime-ameliorated temperatures reduced the effects of drought stress on coastal vegetation. To test this, weekly measurements of water potential and stomatal conductance were made on two coast evergreen tree species, *Arbutus menziesii* and *Quercus agrifolia*, throughout the summer 2014 dry season. Water potential remained generally constant during this period but stomatal conductance declined in both species as the dry season progressed. Species' resistance to embolism was determined using the centrifuge method, and showed *Q*. *agrifolia* to be more vulnerable to embolism than *A*. *menziesii*. The stem vulnerability curves were consistent with species' seasonal water relations as well as their anatomy; the ring-porous *Q*. *agrifolia* had substantially larger conduits than the diffuse-porous *A*. *menziesii*. Leaf turgor loss points differed significantly as did other pressure-volume parameters but these data were consistent with the trees' seasonal water relations. Overall, the two species appear to employ differing water use strategies; *A*. *menziesii* is more profligate in its water use, while *Q*. *agrifolia* is more conservative, with a narrower safety margin against drought-induced loss of xylem transport capacity. Despite the extended drought, these species exhibited neither branch die-back nor any obvious symptoms of pronounced water-stress during the study period, implying that the maritime climate of California's central coast may buffer the local vegetation against the severe effects of prolonged drought.

## Introduction

**Fig 1:**
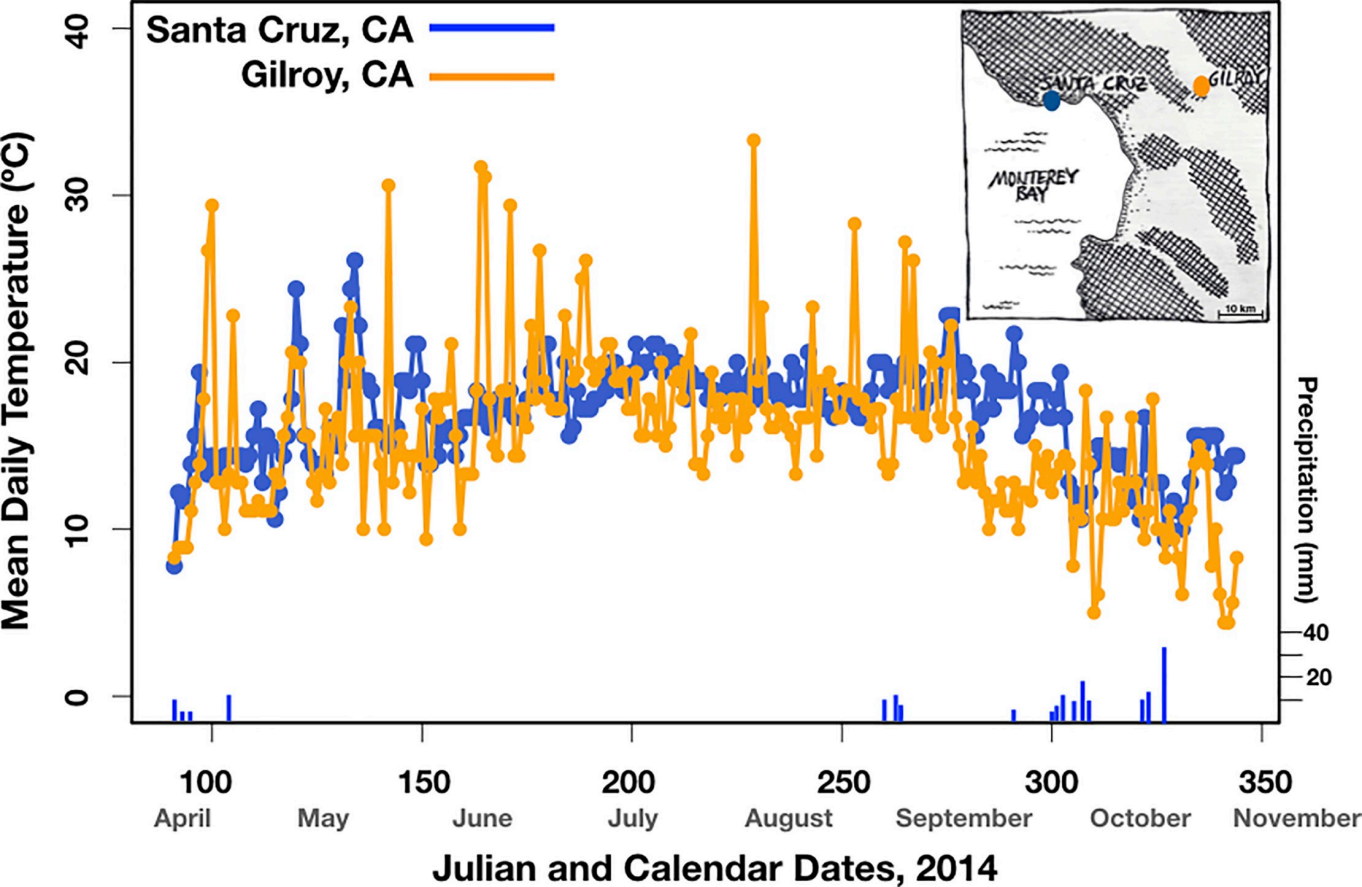
The mean daily temperature in Santa Cruz and Gilroy, California from April to November, 2014. Precipitation data are shown for Santa Cruz only; Gilroy received slightly less rain. The maritime influence buffered the temperature in Santa Cruz relative to Gilroy.

Prolonged drought can lead to plant mortality. Water deficit can induce stress by carbon starvation, failure to transport water through the xylem tissue, or indirectly, by increasing plants’ vulnerability to biotic agents [1] [2]. Recent studies have shown that xylem failure is the main driver of drought-induced plant mortality [2]. Critically important to plant survival, the xylem tissue balances the need for water transport with safety against drought-induced air entry (embolism) depending on the plant's life history strategy and habitat. However, many tree species, angiosperms in particular, appear to maintain narrow safety margins against embolism, and are therefore sensitive to changes in water availability [3]. Plants in Mediterranean regions, such as coastal California, are adapted to extended dry seasons followed by wet winters, but years with high temperatures and markedly reduced winter rains (e.g. 2011–2015; Fig 1), can have a profound effect on plant growth and fitness [4] [5]. Indeed, California’s changing climate has led to mortality and demographic shifts in forest trees [6] [7] and even the xeric-adapted chaparral ecosystems [4]. In coastal areas, summer fog and the maritime influence provide a buffer against extreme evaporative stress and high summer temperatures [8] [9] [Fig 1], but the degree to which plants in these regions can tolerate prolonged water stress remains unclear. Here, we examined the drought tolerance and seasonal water status of two co-occurring evergreens on California's central coast, Pacific madrone (*Arbutus menziesii*, Ericaceae) and coast live oak (*Quercus agrifolia*, Fagaceae) to assess the degree to which the 2011–2015 drought affected the physiology of these species.

*A*. *menziesii* and *Q*. *agrifolia* are common evergreen broadleaf sclerophyllous trees found in coastal and inland California. *A*. *menziesii* has a distribution extending from pockets in central and southern California to a continuous range from the San Francisco Bay up to the coast of British Columbia, whereas *Q*. *agrifolia* occurs from upper Baja California to just past the San Francisco Bay. These species make an interesting comparison in the Monterey Bay area because this region comprises the southern range limit of *A*. *menziesii* and the northern range limit of *Q*. *agrifolia*. While *A*. *menziesii* is the more northerly distributed species, and presumably the more mesic adapted, it is still commonly considered to be drought resistant. Madrones access ground water with deep taproots, which have been recorded to penetrate bedrock deeper than 3.5m [10]. They also possess diffuse-porous wood anatomy, with regularly arrayed, relatively small conduits at high density, an arrangement commonly associated with embolism resistance [11]. In contrast, *Q*. *agrifolia* is may be more shallow-rooted [12] [13], it exhibits more ring-porous anatomy, with fewer, but larger earlywood conduits that are more vulnerable to embolism at the onset of drought (or freezing) [14] [15] [16]. Studies show that *Q*. *agrifolia* has vasicentric tracheids, which may assist in keeping large vessels hydrated during periods of water shortage [17] [18]. *Q*. *agrifolia* is found throughout central and southern California, it is highly water-use efficient, and it responds rapidly to rain by increasing sap flow and photosynthesis [19].

To gain a better understanding of the potentially differing water use strategies of these two anatomically distinct yet co-occuring species, we evaluated their level of water stress and stomatal conductance over the course of the summer and autumn growing season of 2014. These data are contextualized with lab-based measures of xylem vulnerability to embolism and leaf turgor loss point. In light of the warmer temperatures and uncertain precipitation patterns facing future California, studies such as ours are especially pertinent for evaluating these species' long term performance in a region that is likely to become progressively drier over the next century [7].

## Materials and methods

### Study site and sampling

The bulk of the study was conducted in 2014 at the University of California, Santa Cruz (UCSC). The campus is on the north shore of Monterey Bay in central California, on the Pacific side of the Santa Cruz coast mountain range. The region experiences a mediterranean climate characterized by dry, foggy summers followed by winter rains but the regional orography and proximity to the ocean create weather that is considerably cooler and wetter than that of inland valleys such as in Gilroy (data from the National Oceanographic and Atmospheric Association; Fig 1). The 2011–2015 drought was atypical so precipitation during this time was neglible in both localities until day 329, after which climate stations registered meaningful rain events.

The study was performed at the UCSC Forest Ecology Research Plot (UCSC-FERP; [20]). The site is a 6 ha mapped forest dynamics plot of mixed evergreen coastal forest (37°0.745’N, 122°4.490’W), with an elevation of 314 to 332 m. It is within a 10 min drive to campus. Individuals of *A*. *menziesii* and *Q*. *agrifolia* on the plot were sampled along a 200 by 60 m east-west transect to control for elevation.

### Seasonal water potential and stomatal conductance

Seasonal measurements of water potential and stomatal conductance were made weekly from April 18th to November 21st of 2014, except the weeks of August 10th, August 18th, and September 21st. All measurements were made between 12:00 and 14:00. The study period captured part of California’s current record drought cycle, and includes the start of a heavy storm event from late November to December. Stomatal conductance was measured on south-facing foliage using a Decagon Leaf Porometer (SC-1 Leaf Porometer; Decagon Devices Corp., Pullman, WA). After measuring stomatal conductance, the leaves (or small branch tips in the case of *Q*. *agrifolia*) were subsequently removed, sealed in a bag, and placed in a cooler with ice for 60–90 minutes prior to transport to the lab. Water potential was then measured with a pressure chamber (PMS 1005, PMS Instrument Co., Corvallis, OR). Three to six individuals of *A*. *menziesii* and three to four individuals of *Q*. *agrifolia* were randomly sampled for each midday measurement. This method of measuring water potential was necessary because UCSC-FERP is unusually dense with poison oak (*Toxicodendron radicans*), the oils of which can easily contaminate tarps and equipment and cause severe allergic reactions. We tested the instantaneous leaf water potentials against paired leaves left to equilibrate in a cooler for 60–90 minutes and found no significant difference in neither the means nor the variance (n = 9–10 leaves per treatment from five campus trees; p = 0.19).

### Diurnal measurements

Diurnal water potential (ψ) and stomatal conductance (*g*_*S*_) measurements were conducted for *A*. *menziesii* on April 18th, June 24th, and October 3rd; and for *Q*. *agrifolia* April 20th, June 26th, and October 5th. Data were recorded hourly from pre-dawn to sunset. Each hour, three trees were selected randomly (from the approximately thirty trees on the plot), and stomatal conductance was measured on a sun exposed branch. The leaf (or branchlet for *Q*. *agrifolia*) was then removed and sealed in a plastic bag. The bagged samples were allowed to equilibrate in an ice cooler for 15 minutes before each water potential measurement.

### Maximum conduit length

Maximum conduit length was determined for each species following the methods of Greenidge (1952; [21]). Six branch samples (ca. 2 m long) of each species were cut from the trees and transported to the laboratory in plastic bags. For each branch, the distal end was removed, trimmed with a razor blade and connected to pressurized air (approximately 0.1 MPa). Then, the base of the branch was trimmed underwater in increments of ca. 3 cm until air bubbles appeared from the cut end. The length at which bubbles appeared was taken to be the maximum vessel length of the sample.

### Vulnerability curves

Vulnerability curves were generated using the centrifuge method [22]. Segments longer than the maximum vessel length (*A*. *menziesii*, 0.29 ± 0.05 m; *Q*. *agrifolia*, 0.89 ± 0.22 m; 5–7 mm in diameter) were excised underwater from trees using a modified funnel sealed with plumbers putty. These were transported to the lab in humidified plastic bags and there trimmed under water to the measurement length of 0.142 m. The samples were then submerged under de-ionized filtered water, and vacuum (ca. 0.05 MPa) was applied overnight to reverse any embolism. The following morning, the maximum conductivity was measured (K_max_), and then the samples were centrifuged in a custom rotor to induce xylem tension [22] [23]. After each centrifuge spin, the conductivity (K_x_) was measured and the percent loss conductivity (PLC) was calculated at each pressure as (1- K_x_/K_max_)*100. Samples were spun for 4 minutes in increments of 0.5 MPa until all the stems reached less than 10% of maximum conductivity. The data were fitted with a non-linear regression function. Due to sampling constraints, samples for the vulnerability curves were taken from trees directly adjacent to the UCSC-FERP in mid-June 2014. Six stems for each species of similar DBH to those sampled on the plot were selected, and water potential at the time of harvest was measured to ensure similar hydraulic conditions.

Concerns over the effect of vessel length on centrifugal measures of xylem vulnerability to embolism [24] [25] prompted us to repeat the above experiment in 2019 with *Q*. *agrifolia* stems using a rotor designed to fit segments that were 252 mm in length. This rotor is substantially heavier and imposes more strain on the centrifuge motor than the 142 mm model, so the experiments were performed as above but spun in 1 MPa increments to reduce wear and tear. We constructed the vulnerability curve per the methodological recommendations described by Jacobsen and Pratt, 2012 ([26], see also [23]); these studies have shown that keeping the cut ends wet while in and outside of the rotor is critical for minimizing long-vessel artefacts.

Given the potential problems associated with long vessel effect, we verified the oak vulnerability curves by measuring native PLC's in *Q*. *agrifolia* toward the end of the growing season when conditions are driest. Eight stem segments exceeding 2 m in length were cut at pre-dawn hours because relaxed sap tensions are thought to minimize the potential of the 'cutting artefact' [27]. The stems were wrapped and sealed in two extra-large plastic bags and brought to the laboratory where water potentials were allowed to equilibrate for at least 60 min. Subsequently, two to three leaves were selected for measures of leaf water potential, and when no significant water potential differences were discernible between these leaves, the stem was prepared for hydraulic measures. Stem segments measuring over 1.1 m in length and 5–11 mm in diameter (thus comparable in girth to the spun segments) were cut under water and fitted to a standard hydraulic apparatus as described above to measure native conductivity (Kn). The segments were subsequently flushed at 100 kPa with filtered water, and the maximum conductivity was measured (K_max_). The PLC was computed as described above.

Growth rings are difficult to discern in *Q*. *agrifolia* (see Fig 6) so it is likely that the previous year's xylem was included in the measurements of K_max_. Earlier work has shown that the inclusion of older, previously embolized xylem in conductivity measures may inflate K_max_, and thus overestimate PLC [28]. We stained the samples with crystal violet in an attempt to quantify the functional xylem [29], expecting that older vessels would no longer be functional. However, we were unable to identify any clear patterns, seeing instead that in some samples older xylem harbored functional vessels of all sizes, whereas in other samples patches of xylem were dysfunctional irrespective of xylem age. Some degree of caution is always warranted, but given the heterogeneity of our observations, it seems reasonable to conclude that our approach adequately captures the *in situ* hydraulic response of *Q*. *agrifolia*.

### Estimated seasonal percent loss of conductivity

The percent loss of conductivity at a given water potential was estimated for each species throughout the summer using the vulnerability curve fit. At each sampling point, the PLC was calculated from the non-linear regression using the mean, minimum, and maximum water potential at that time. Refilling was presumed not to occur and therefore PLC could only increase over the course of the growing season (i.e. whenever the xylem tension decreased, PLC was kept constant from the previous sampling period).

### Xylem anatomy

The stem segments used for vulnerability curves were subsequently sectioned at mid-point for anatomical measurements. Xylem conduit diameters were measured on a subsample of 4 stems of each species. Transverse stem cross-sections were cut by hand using a razor, and stained in toluidine blue to increase contrast. The sections were photographed using a compound microscope (Motic BA400; JH Technologies, San Jose, CA) coupled to a digital camera. Digital composite images were generated using the Pairwise Stitching function in Fiji [30], and analyzed using ImageJ [31]. Lumen conduit diameter was computed by treating the lumen areas as circles; in oaks lumen diameters below 10 μm were not included in the analysis as they often numbered in the thousands. Hydraulic mean diameter was calculated as D_h_ = D^5^/D^4^ [32].

### Leaf pressure-volume analysis

Pressure volume curves were performed in 2019 to primarily discern differences in osmotic potential at leaf saturation (Ψ_ΠMAX_, MPa), relative water content (RWC) and the leaf turgor loss point (Ψ_TLP_; MPa) between the two species. Leaves were collected on site and transported back to the laboratory in humidified bags and hydrated for ~ 2 hours. Most samples reached water potentials between predawn and midday. Hydrated samples were then alternatively weighed on analytical balance and water potential was measured as they dried. Between measures, samples were placed in dark, sealed bags for about 15 min to equilibrate water content and slow the rate of drying. Each sample was measured 10 to 15 times during dry-down, with at least five measures past the inflection point to capture the linear portion of the curve. After dry-down measures, samples were oven dried at 65°C for ≥ 72 h and then weighed. Samples were inspected for signs of over-saturation by plotting the mass of water loss in relation to leaf water potential. Points with an initial steep decline in water potential were removed to avoid the analysis of excessively hydrated leaves [33]. Relative water content (RWC) was computed according to equation (Eq 1).

$$RWC=\left(W_{f}-W_{d}\right)/\left(W_{s}–W_{d}\right)$$ (1)

Where W_f_ is fresh weight (g), Wd is dry weight (g), and W_s_ is saturated weight (g) of the leaf sample. Saturated leaf weight was taken as the intercept of the initial slope—above the inflection point—where leaf water mass corresponded with a water potential of 0 MPa.

Analyses of turgor loss point and osmotic potential were performed by plotting the relative water deficit (RWD; 1 –RWC) and inverse water potential. The last three points beyond the inflection point were fitted with a linear regression, points were then added towards the inflection point until the goodness of fit was maximized. The water potential at which the linear slope intercepts the inverse water potential axis was the maximum leaf osmotic potential (Ψ_ΠMAX_). To find the water potential at the turgor loss point (Ψ_TLP_), we found the point that best occupied the transition of the linear portion of the data to the curve, using the linear regression. The slope of the turgor pressure in relation to RWC represented the leaf modulus of elasticity (ε).

### Isotope analysis

Ten leaves from each of six trees per species were collected for isotope analysis on Nov. 23^rd^, 2014. The samples were oven dried, ground and pooled, and analyzed at the Institute of Marine Science Analytical Laboratory at the University of California Santa Cruz for δ^13^C composition and C:N ratios using a Thermo ElementXR High Resolution Inductively Coupled Plasma Mass Spectrometer (ThermoFisher Scientific.com).

### Statistical analysis

All analyses were performed in the R environment [34]. The Shapiro-Wilk test (shapiro.test) assessed normality and a var.test evaluated sample differences in variance. A standard Welch's t-test function (t.test) evaluated differences between sample means for normally distributed data, otherwise a Wilcoxon test (wilcox.test) was used. All data are reported as averages ± 1 SD, with an α = 0.05. Sample sizes are listed in the figure captions and Table 1.

**Table 1:** Means, standard deviations, and P-values for traits derived from leaf pressure-volume curves, hydraulic and anatomical measures. The sample size is n = 6 for all traits except vessel density, where n = 4.

	*Quercus agrifolia*	*Arbutus menziesii*	*p-value*
Water potential at the turgor loss point ψ_TLP_ (MPa)	-2.58 ± 0.5	-1.91 ± 0.57	P = 0.026
Modulus of elasticity, ε (MPa)	30.58 ± 8.38	15.14 ± 3.44	P < 0.001
Osmotic potential at full turgor, π (MPa)	-2.15 ± 0.65	-1.26 ± 0.49	P = 0.008
Relative water content at the turgor loss point, RWC (%)	92.8 ± 2.29	91.98 ± 2.32	P = 0.49
Huber value (wood:leaf area)	3.92e-0.5 ± 8.32e-06	3.62e-05 ± 2.03e-0.5	P = 0.24
Specific Conductivity (K_s_, kg MPa^-1^ s^-1^ m^-1^)	2.29 ± 1.06	2.06 ± 0.41	P = 0.64
Vessel Density (vessels m^-2^)	9.25e+07 ± 7.45e+07	4.73e+08 ± 6.74e+07	P < 0.001
Leaf δ^13^C values (‰)	-31.47 ± 0.84	-31.85 ± 0.61	P = 0.4

## Results

**Fig 2:**
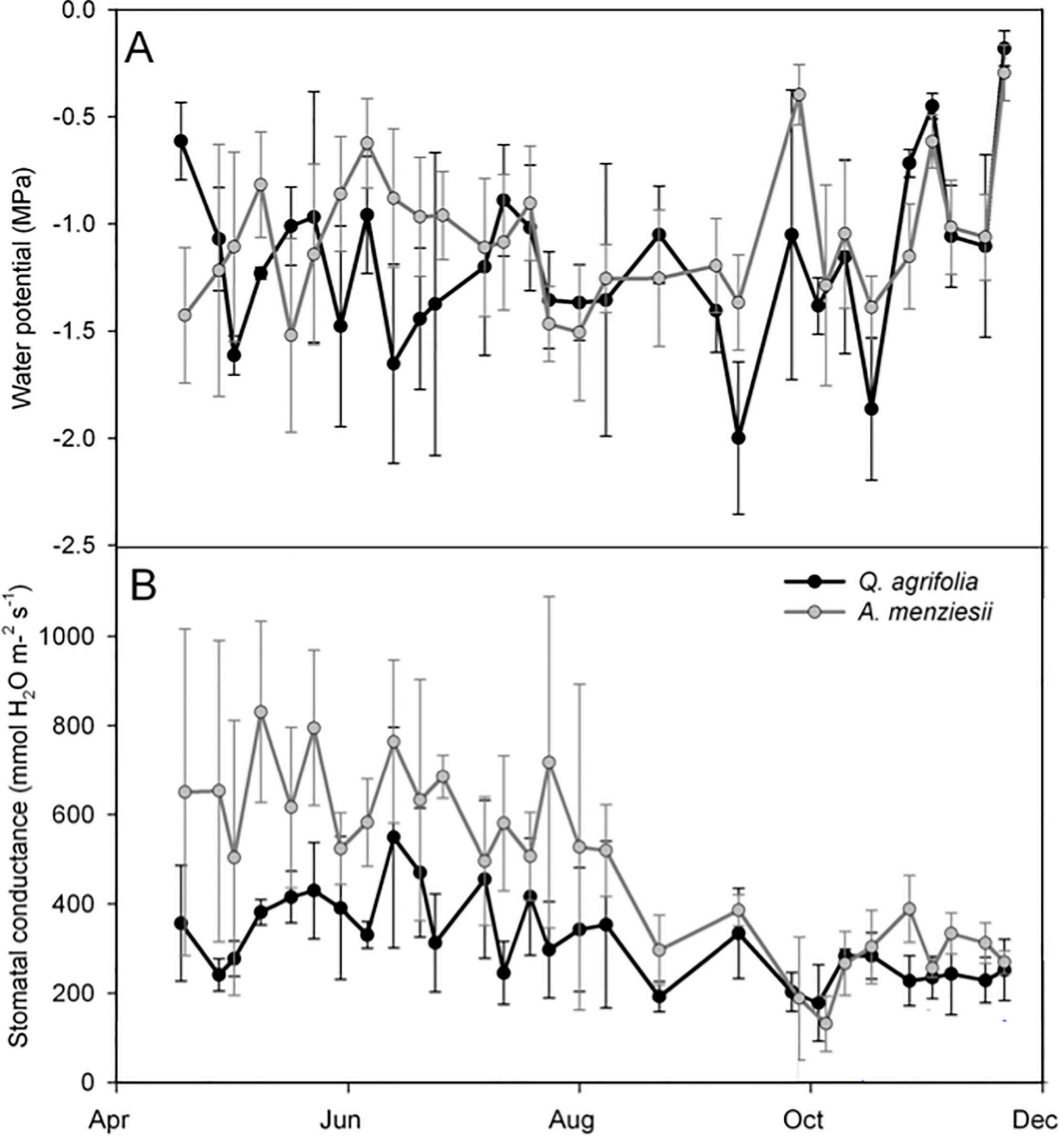
Seasonal mid-day water potential and stomatal conductance measures for *Q*. *agrifolia* and *A*. *menziesii* during the 2014 summer and fall season in 2014. Sample size was n = 3–6 leaves per species per day, although n = 4 leaves for the majority of the measures.

Over the course of the sampling period, the midday water potential of both species was highly variable (Fig 2A), showing no apparent seasonal trend until the return of rain in autumn when both species showed a recovery to more moderate values. Minimum water potentials (Ψ_min_) during the summer season fell to -2.5 MPa and -1.87 MPa in the oak and madrone respectively, with maximum values peaking at -0.092 MPa and -0.158 MPa with the start of the rains in October. Stomatal conductance in *A*. *menziesii* was consistently, though not significantly higher than that of *Q*. *agrifolia* during most of the sampling period but converged to values between 200 and 400 mmol m^-2^ s^-1^ starting in October (Fig 2B).

**Fig 3:**
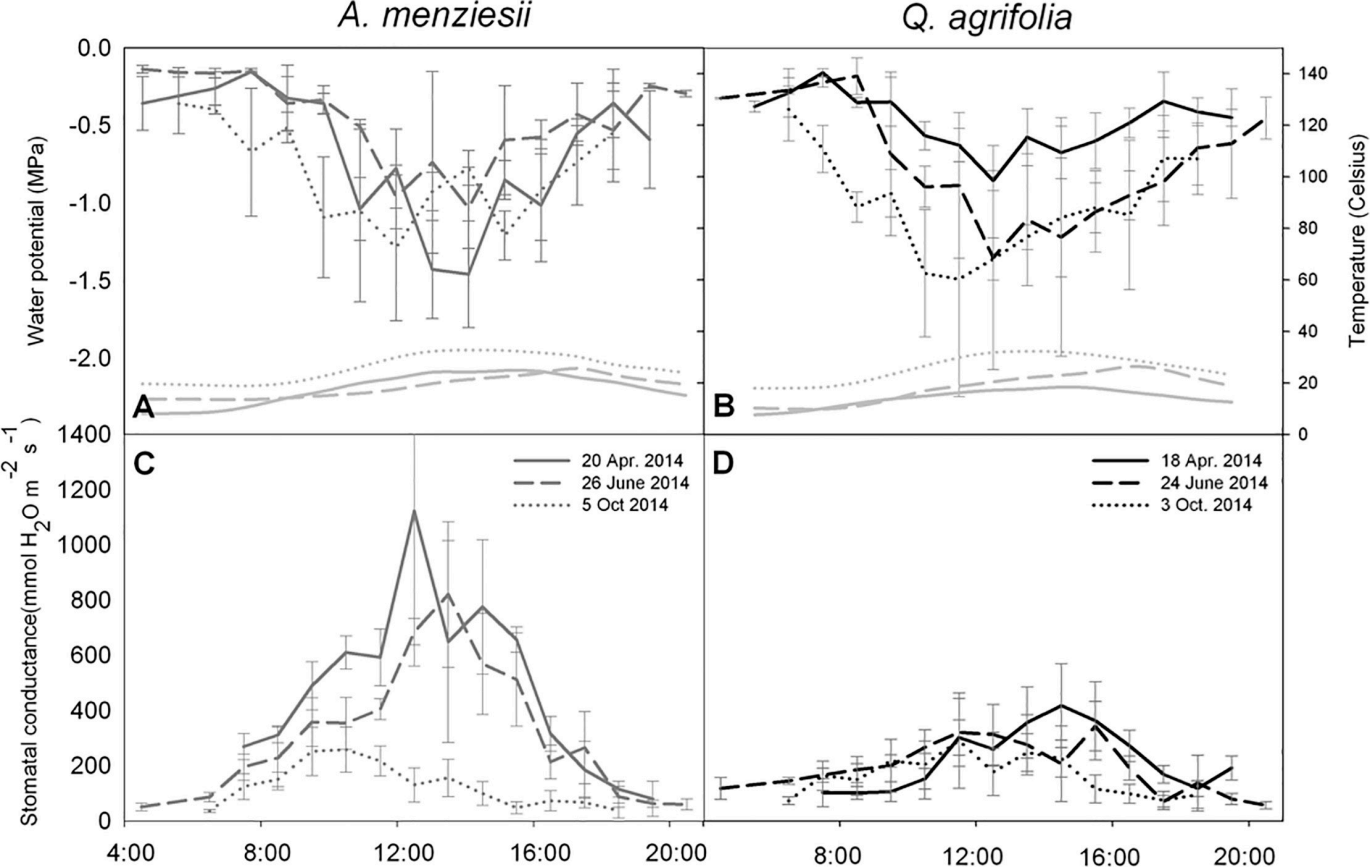
Seasonal diurnal measures of water potential and stomatal conductance in *Q*. *agrifolia* and *A*. *menziesii*. N = 3 leaves per species per hour.

Diurnal measurements of water potential and stomatal conductance provided further insight into the changing water relations of these species over the dry season. *A*. *menziesii* showed the lowest water potentials after 12:00 and there was no obvious difference between diurnal water potentials as the dry season progressed (Fig 3A). By contrast, diurnal water potentials not only declined over the dry season in *Q*. *agrifolia* but reached their low points earlier in the day (Fig 3B). In both species, stomatal conductance peaked between 12:00 and 16:00 (Fig 3C and 3D) but whereas conductance dropped substantially in *A*. *menziesii* over time, it remained largely unchanged in *Q*. *agrifolia*, consistent with the seasonal measures shown in Fig 2.

**Fig 4:**
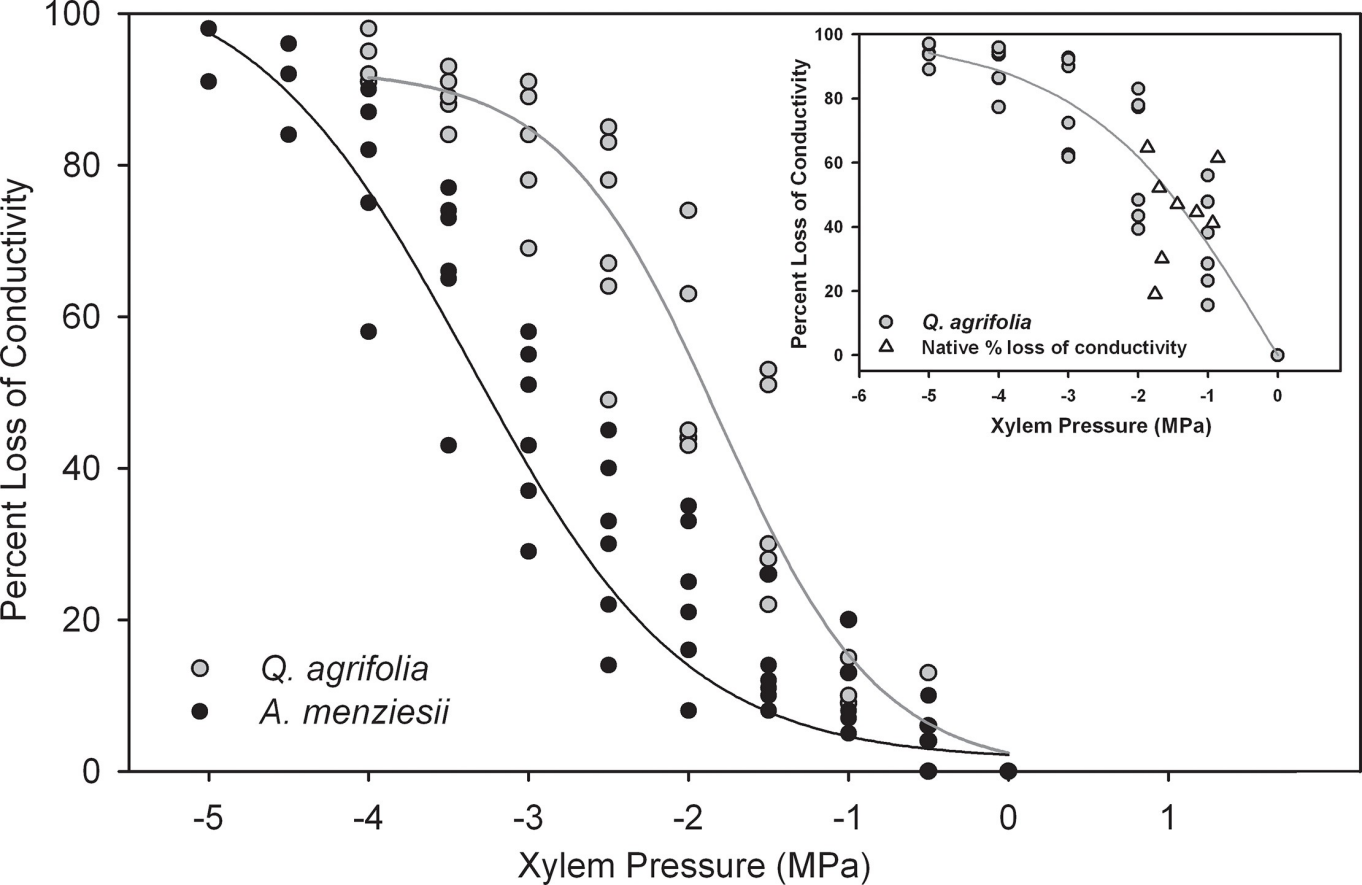
The percent loss of conductivity as a function of xylem pressure (vulnerability curve) in 14.2 cm stems of *Q*. *agrifolia (*P_50_ = -1.96 ± 0.32 MPa) and *A*. *menziesii* (P_50_ = -3.0 ± 0.33 MPa; n = 6 per species). The inset shows the vulnerability curve on 25.2 cm stems of *Q*. *agrifolia* (P_50_ = -1.79 ± 0.67 MPa; n = 6) as well as the native PLC values for this species.

While *A*. *menziesii* appeared to be the more profligate water user, it was still more resistant to embolism than *Q*. *agrifolia* (Fig 4), with a P_50_ of -3.0 ± 0.33 MPa compared to -1.96 ± 0.32 MPa for *Q*. *agrifolia*. In both species, embolism showed a sigmoid response to water stress, but given that vessels were substantially longer in *Q*. *agrifolia*, we constructed a second vulnerability curve from stems measuring 25.2 cm in length (Fig 4, inset) to test if a long-vessel artefact skewed our data. The 25.2 cm oak stems produced vulnerability curves with a P_50_ of -1.79 ± 0.67 MPa, which is statistically indistinguishable from the mean P_50_ value produced by the shorter stems (t-test; P = 0.59). In addition, we observed no significant differences in either the P_88_ or the slope of the two vulnerability curves. The P_12_ could not be reliably computed from the second vulnerability curve. The native PLC measures in *Q*. *agrifolia* were consistent with the vulnerability curves: water potentials and PLCs ranged from -0.86 MPa to -1.87 MPa, and 19% to 64% (Fig 4, inset).

**Fig 5:**
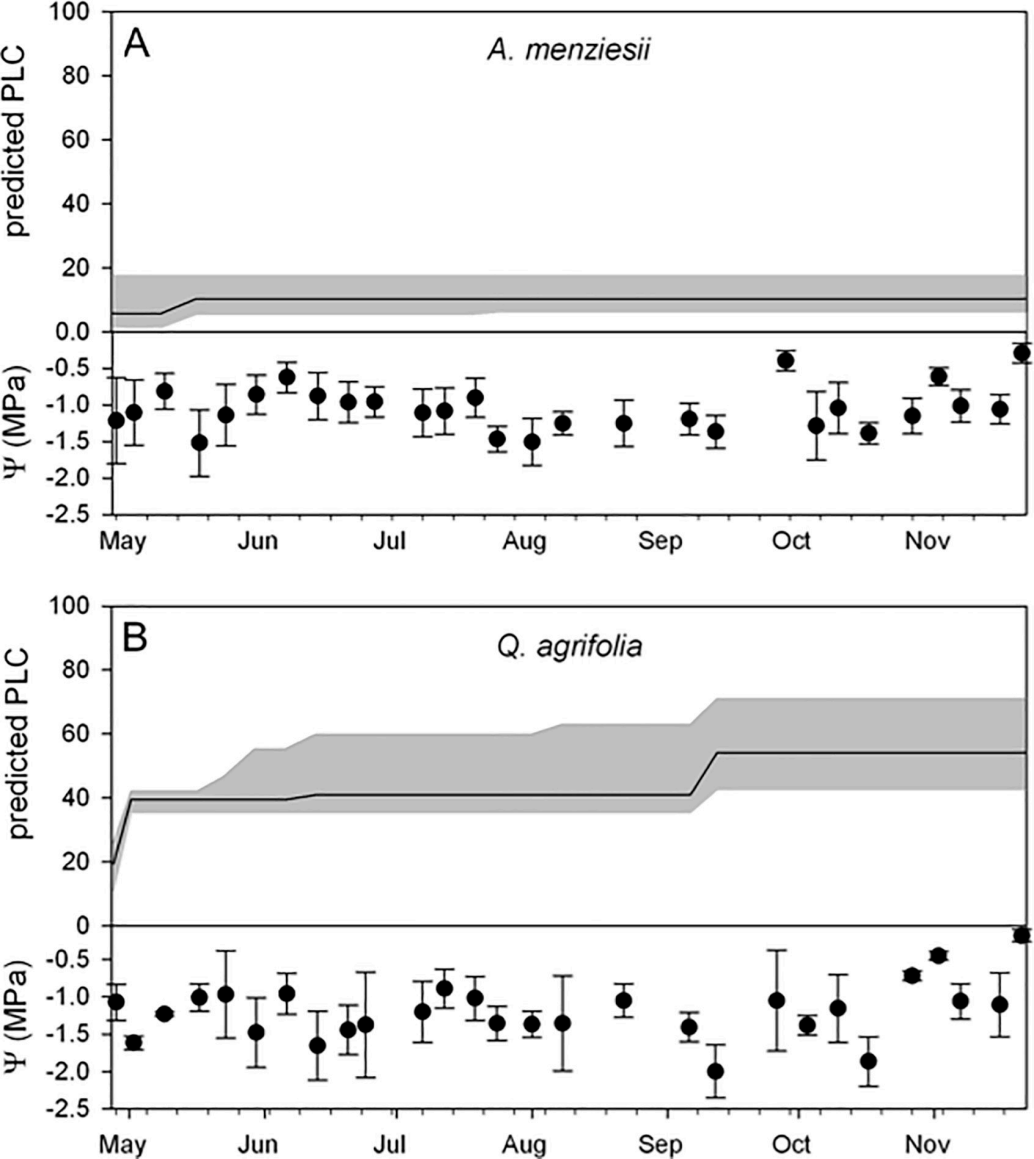
Mean (in black) and minimum to maximum range (in gray) predicted percent loss of conductivity (PLC) for *A*. *menziesii* (A) and *Q*. *agrifiolia* (B) over the measurement period. The predictions are based on the mean, minimum and maximum water potential recorded at each time point and the mean PLC response of the species from the vulnerability curves, assuming no recovery from embolism. The bottom of each panel indicates the mean ± SD of the water potential measures at each time point during the season.

Modelled estimates of seasonal embolism suggest that *A*. *menziesii* experiences a minor loss of hydraulic conductance over the dry season, consistent with its relatively well-hydrated state during the sampling period (Fig 5A). By contrast, the ring-porous *Q*. *agrifolia* is likely to suffer over 50% embolism by the end of the summer season (Fig 5B).

**Fig 6:**
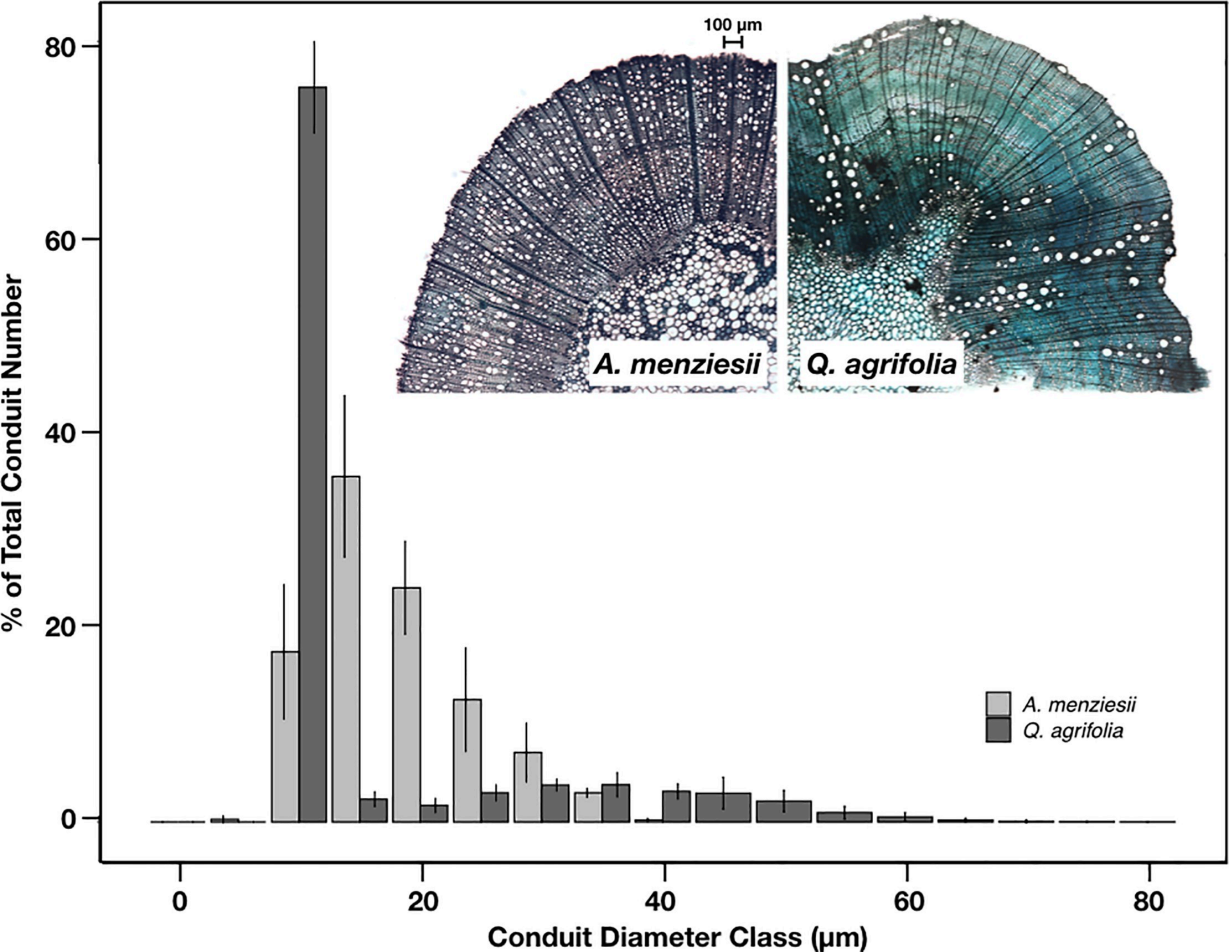
Vessel diameter distributions in stems of *Q*. *agrifolia* and *A*. *menziesii* (n = 4 stems per species). Inset micrographs show the typical stem xylem structure of these two species.

The two species’ xylem anatomy differed substantially (Fig 6). Xylem in *A*. *menziesii* is diffuse porous, with many small conduits of similar size (mean diameter = 20.96 ± 6.26 μm, hydraulic mean diameter = 28.1 ± 2.0 μm), while *Q*. *agrifolia* is ring porous, with larger more isolated vessels and greater variation in vessel (and potentially tracheid) diameter (mean diameter = 38.1 ± 11.9 μm, hydraulic mean diameter = 48.0 ± 5.4 μm). Vessels in *Q*. *agrifolia* were also longer than *A*. *menziesii*, with a maximum vessel length of 0.89 ± 0.22 m compared to 0.29 ± 0.05 m for *A*. *menziesii*. Despite these differences, both species had similar specific conductivities and wood to leaf area ratios, suggesting that the greater diameter and length of the vessels in *Q*. *agrifolia* adequately compensated for their lower vessel density (Table 1).

The leaf water relations of both species differed in ways that corresponded with their seasonal water potentials. The water potential in *Q*. *agrifolia* at the leaf turgor loss point was -2.58 ± 0.5 MPa, perilously close to the minimum seasonal water potential of -2.5 MPa, while the Ψ_TLP_ in *A*. *menziesii* was -1.91 ± 0.57 MPa, which again was just slightly below the Ψ_min_ of -1.87 MPa (Table 1, Fig 2). This suggests that leaves of both species may have lost some turgor during the most stressful time of the growing season (August, Fig 2). The relative water content at the turgor loss point (RWC_TLP_) was nearly identical in both species at 92–93% but achieved by different means: *Q*. *agrifolia* had lower osmotic potential at full turgor (π) than *A*. *menziesii* (-2.15 ± 0.65 MPa and -1.26 ± 0.49 MPa respectively) and stiffer leaf tissues as estimated by the modulus of elasticity (ε), which was 30.58 ± 8.38 MPa for *Q*. *agrifolia* and 15.14 ± 3.44 MPa in *A*. *menziesii* (Table 1). In other words, relative to *Arbutus*, *Quercus* maintained turgid leaves by investing into osmotically active sugars and solutes in combination with stiffer cell walls.

Lastly, both species had nearly equivalent leaf δ^13^C values spanning -30.31‰ in *Quercus* to –32.7‰ in *Arbutus*, a range indicative of relatively high leaf intercellular CO_2_ levels, and from a integrated seasonal perspective, practically no effects of water stress.

## Discussion

The drought conditions of 2014 were the most arid in California over the past 1200 years [35] and while massive tree dieback was observed in the central and Sierra Nevada region, the coast live oaks and Pacific madrones at our study site experienced little, if any drought stress. Rather, our data indicate that the marine-moderated climate may have partially buffered *A*. *menziesii* and *Q*. *agrifolia* against climatic extremes. In both species, water potentials rarely dipped below -2.5 MPa and while stomatal conductance in *Q*. *agrifolia* remained relatively low over the growing season, *A*. *menziesii* did not show a notable drop in stomatal conductance until August. Diurnal measures of water potential and *g*_*S*_ reinforced these trends as did measures of leaf carbon isotopes. *Arbutus'* deep roots may provide it with a notable advantage during the dry, late-season summer months. Embolism resistance in both species was consistent with their seasonal water potentials although the diffuse-porous *A*. *menziesii* had a larger hydraulic safety margin, that is the difference between the P_50_ and the seasonal minimum water potential [3]. On the whole, the water relations of these two evergreens are consistent with the water availability in their native habitat, even during a period of exceptional drought.

Seasonal as well as diurnal measures of leaf water potential and stomatal conductance suggest that both species exerted a higher degree of stomatal control over water loss as the season progressed. However, the water-use strategy of these species differed considerably. For example, stomatal conductance in *A*. *menziesii* was relatively high, often double that of *Q*. *agrifolia*, but by October 2014 its *g*_*S*_ was reduced to only 23% of what it was in spring (Fig 2). By contrast, *g*_*S*_ in *Q*. *agrifolia* was low and steady during the growing season relative to *A*. *menziesii*, with the oaks retaining 70% of their maximum stomatal conductance well into October. Simply put, *A*. *menziesii* is a water spender that exerts strong stomatal control at the end of the growing season, whereas *Q*. *agrifolia* is a consistently moderate water consumer over the course of the year. Mid-day water potential varied considerably in both species, largely tracking water availability as seen in October. Shifting water potentials coupled with different stomatal strategies complicate any simple categorization of these species as isohydric or anisohydric, as discussed by Hochberg et al. (2018; [36]).

The relative impact of water stress on the xylem of the two species is consistent with their anatomy. Large-vesseled species are generally more susceptible to embolism [11], so per expectation, the xylem of the large-vesseled *Q*. *agrifolia* was more vulnerable to air entry than *A*. *menziesii*. These two species represent the opposite ends of the xylem anatomical spectrum for evergreen angiosperms in central California. *Q*. *agrifolia* is effectively ring-porous, with a small number of large conduits and has a relatively shallow rooting depth [12] [13], while *A*. *menziesii* is diffuse porous, with many smaller conduits, and is deeply rooted [10] [37]. Vulnerable xylem may explain why *Q*. *agrifolia* was predicted to experience a considerable amount of embolism by the end of the summer relative to *A*. *menziesii*, whereas a combination of smaller, shorter and more numerous vessels may explain *A*. *menziesii*'s higher resistance to embolism. Specific conductivity (K_s_) was similar in both species despite the differences in xylem anatomy; higher vessel density in *A*. *menziesii* compensated for smaller vessel size.

The degree to which long vessels may affect measures of vulnerability to cavitation in oaks and lianas has been the subject of considerable debate. The centrifuge method is at the center of the dispute with studies showing that during spins, water can be pulled out of vessels that are longer than the rotor because removing the end-walls creates a capillary effect [38] [25] [39]. However, this appears to be a particular problem when flow is measured during centrifugation rather than after the spin [40] [41] [42] [43]. Some studies show that stems (or roots) appear progressively more vulnerable as segment length is reduced and the number of open vessels increases, producing an r-shaped curve [40]. However, a number of studies counter these findings, showing that segments of different lengths have similar P_50_ values if precautions are taken, such as keeping the ends of the stem wet during the spin [26] [41–43]. Interestingly, Barotto et al. (2016) report an r-shaped curve for a *Eucalyptus* species with short vessels, whereas other eucalypts with longer vessels produce sigmoidal curves [18]. In any case, vulnerability curves from *Q*. *agrifolia* stems measuring 25.2 cm and 14.2 cm in length were identical, with the shorter stems producing a clear sigmoidal curve (Fig 4). Our results are supported by native PLC data, which are consistent with the PLC values predicted by the curves, as well as the observed range of seasonal water potentials, which by extension suggest that approximately 50% of stem hydraulic conductivity remains after the summer dry season (Fig 5).

Any number of available methods are currently being used to generate vulnerability curves [44] [45] [46], some of which may yield different P_50_ values on the same set of species. For example, Jacobsen et al. (2007) used the dehydration method to produce stem *Q*. *agrifolia* vulnerability curves with nearly identical P_50_ values to those in Fig 4 [47], whereas the optical curves produced by Skelton and colleagues yielded P_50_ values of −4.32 ± 0.26 MPa in the leaves and −4.45 ± 0.24 MPa in stems [48]. It seems reasonable to propose that phenotypic plasticity or regional population differences explain these different P_50_ values although Skelton et al. (2019) report no regional variation in *Q*. *douglasii* [49]. In any case, it would be interesting to apply the optical method to the coastal assortment of conifer and angiosperm evergreens to better understand the extent to which hydraulic segmentation operates in plants from marine-ameliorated systems; embolism resistance often incurs carbon costs [50] so selection may favour more vulnerable leaves and stems in coastal versus inland populations.

The difference between species' P_50_ and the seasonal minimum water potential is known as the hydraulic safety margin, and is broadly accepted as an indicator of species' susceptibility to drought [3]. With a P_50_ of -1.79 ± 0.32 MPa and a minimum seasonal water potential of -2.5 MPa, *Q*. *agrifolia* exceeded its P_50_ by 0.71 MPa, potentially operating slightly beyond its safety margin for several days during 2014, with possible loss of leaf turgor (Ψ_TLP_ in this species is -2.58 ± 0.5 MPa). Embolism repair has been reported to occur in *Quercus gambelii* [16] [41], potentially counteracting the frequent loss of conductivity in larger vessels, but whether refilling occurs in other oak species is poorly understood. By contrast to the oak, *A*. *menziesii* maintained a safety margin of 1.13 MPa given that it's Ψ_min_ reached only -1.87 MPa, whereas its P_50_ and Ψ_TLP_ were -3.0 ± 0.33 MPa and -1.91± 0.57 MPa, respectively. Of the two species, *Q*. *agrifolia* is more vulnerable to water stress, but given the ameliorated climate of its coastal habitat, it is not surprising that it maintained favourable levels of hydration even during one of the most severe droughts in California's history. Much of Santa Cruz county stands on sandy marine terraces prone to sinkholes [51] so it may be that the roots of both of these evergreens tap into more permanent underground water to remain hydrated. Indeed, deep roots in *A*. *menziesii* may help explain its extravagant use of water from April to August 2014.

Taken together, our data show that coastal *A*. *menziesii and Q*. *agrifolia* and suffered little to moderate water stress during the height of the 2011–2015 California drought. It should be noted, however, that after several years of high temperatures and little rain, trees of neither species appeared particularly healthy. Seasonal growth was limited each spring to a few fresh leaves and even more worrisome was the susceptibility of these trees to insects and molds. Indeed, *Q*. *agrifolia* trees were devastated in 2014 by the california oak moth (*Phryganidia californica*) presumably because years of low carbon uptake left the oaks with reduced carbohydrate stocks for the production of defensive compounds [1]. Longterm annual sampling of California evergreens in both coastal and inland localities could better identify the physiological lesions associated with acute and persistent drought, as well as modes of recovery following episodes of rain.

## Supporting information

S1 Dataset (XLS)
